# Metabolic compartmentalization in the human cortex and hippocampus: evidence for a cell- and region-specific localization of lactate dehydrogenase 5 and pyruvate dehydrogenase

**DOI:** 10.1186/1471-2202-8-35

**Published:** 2007-05-23

**Authors:** Jocelyn D Laughton, Philippe Bittar, Yves Charnay, Luc Pellerin, Enikö Kovari, Pierre J Magistretti, Constantin Bouras

**Affiliations:** 1 Department of Psychiatry, Service of Neuropsychiatry, University Hospitals of Geneva, Belle-Idée, Switzerland; 2 Institute of Physiology, Faculty of Medicine, University of Lausanne, Switzerland; 3 Brain and Mind Institute, Ecole Polytechnique Fédérale de Lausanne, University Medical Centre, University of Lausanne, Prilly, Switzerland; 4 Center for Psychiatric Neurosciences, Department of Psychiatry, University Medical Centre, University of Lausanne, Prilly, Switzerland; 5 Bittar Philippe, spéc. FMH psychiatrie et psychothérapie, av. de Miremont1, 1206 Genève, Switzerland

## Abstract

**Background:**

For a long time now, glucose has been thought to be the main, if not the sole substrate for brain energy metabolism. Recent data nevertheless suggest that other molecules, such as monocarboxylates (lactate and pyruvate mainly) could be suitable substrates. Although monocarboxylates poorly cross the blood brain barrier (BBB), such substrates could replace glucose if produced locally.

The two key enzymatiques systems required for the production of these monocarboxylates are lactate dehydrogenase (LDH; EC1.1.1.27) that catalyses the interconversion of lactate and pyruvate and the pyruvate dehydrogenase complex that irreversibly funnels pyruvate towards the mitochondrial TCA and oxydative phosphorylation.

**Results:**

In this article, we show, with monoclonal antibodies applied to post-mortem human brain tissues, that the typically glycolytic isoenzyme of lactate dehydrogenase (LDH-5; also called LDHA or LDHM) is selectively present in astrocytes, and not in neurons, whereas pyruvate dehydrogenase (PDH) is mainly detected in neurons and barely in astrocytes. At the regional level, the distribution of the LDH-5 immunoreactive astrocytes is laminar and corresponds to regions of maximal 2-deoxyglucose uptake in the occipital cortex and hippocampus. In hippocampus, we observed that the distribution of the oxidative enzyme PDH was enriched in the neurons of the stratum pyramidale and stratum granulosum of CA1 through CA4, whereas the glycolytic enzyme LDH-5 was enriched in astrocytes of the stratum moleculare, the alveus and the white matter, revealing not only cellular, but also regional, selective distributions. The fact that LDH-5 immunoreactivity was high in astrocytes and occurred in regions where the highest uptake of 2-deoxyglucose was observed suggests that glucose uptake followed by lactate production may principally occur in these regions.

**Conclusion:**

These observations reveal a metabolic segregation, not only at the cellular but also at the regional level, that support the notion of metabolic compartmentalization between astrocytes and neurons, whereby lactate produced by astrocytes could be oxidized by neurons.

## Background

In 1988, Fox and Raichle observed by positron emission tomography (PET) a mismatch between glucose uptake and oxygen consumption, raising the possibility that aerobic glycolysis, i.e. the nonoxidative consumption of glucose in the presence of oxygen, may occur in the brain during focal physiologic neural activity [1,2]. Further support to this idea was brought by the observation that a lactate peak could be measured during physiological activation by ^1^H-magnetic resonance spectroscopy (MRS) [3,4]. With the 2-deoxyglucose autoradiographic technique, glucose uptake has consistently been observed in the neuropil, i.e. in regions enriched in dendrites, axons and the astrocytic processes that ensheathe synapses, not the cell bodies [5,6].

Since modern imaging techniques such as PET and functional magnetic resonance imaging (fMRI) are being increasingly used for clinical and fundamental biomedical research, it is of interest to understand cellular biochemical events underling observed signals.

These signals have been shown to result from the interactions between different cerebral cells, raising the concept of "neurovascular unit", including neurons, astrocytes and the vascular endothelium, whereby neuronal activity modulates vascular tension and metabolite delivery from the bloodstream [7]. Apparently, the key cell for the control of vascular tension is the astrocyte [8] (for review, see [9]). For these authors, the vascular tonus is regulated via the stimulation of astrocytic glutamate receptors (mGluRs) triggering the release of vasoactive arachidonic acid metabolites. However, various teams [10-13] seem to think that the cytosolic NADH/NAD^+ ^ratio plays a key role in the modulation of vascular tonus. This ratio is though to be in very close equilibrium with the pyruvate/lactate ratio [14] that depends on glycolysis.

Since pyruvate represents the end-point of glycolysis in mammalian cells, our goal in this study was to indirectly investigate its fate by localizing the two major enzymatic components of its energy production pathways, i.e. the pyruvate dehydrogenase complex (PDHC) and lactate dehydrogenase subunit M (LDH-5).

PDHC is a large, highly organized assembly of several different catalytic and regulatory subunits which catalyzes the oxidative decarboxylation of pyruvate to form acetyl-CoA, CO_2 _and NADH. Pyruvate dehydrogenase (PDH) catalyzes the irreversible entry of pyruvate into the tricarboxylic acid cycle and is therefore a marker for oxidative metabolism, whereas lactate dehydrogenase M subunit (LDH-5 subunit) is necessary for glycolysis to occur at high rate with production of lactate [15,16]. Using immunohistochemistry, we seeked to examine their distribution in the human primary visual cortex and hippocampus. In these two regions, 2-deoxyglucose has been shown to accumulate in specific layers, i.e. the hippocampal stratum moleculare [6] and the layer IV of area 17 [17].

## Results

### Specificity of the antibodies

**Figure 1:**
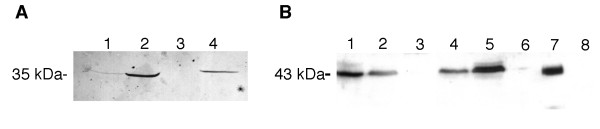
Biochemical characterization of anti-LDH-5 (A) and anti-PDH (B) monoclonal antibodies by SDS-PAGE. 1A) 1, rabbit heart; 2, rabbit muscle; 3, human LDH-1 and 4, human hippocampal extracts. 1B) 1–3, human hippocampal extracts (2, mitochondrial fraction, 3, cytosolic fraction), 4–6, human occipital cortex extracts(5, mitochondrial fraction, 6, cytosolic fraction), 7, PDH from porcine heart and 8, LDH-5 from rabbit muscle (absence of positive band at 35 kDa omitted).

**Figure 2:**
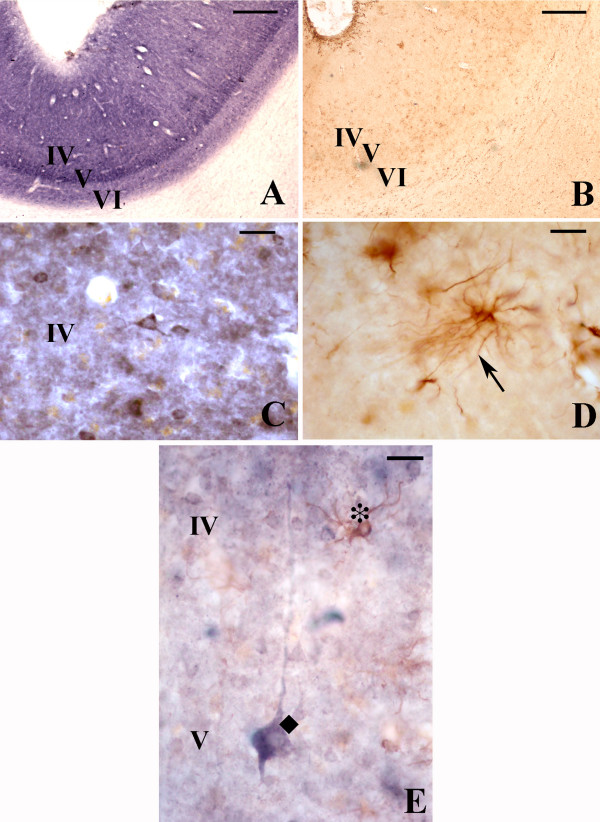
PDH like (A, C) and LDH-5 like (B, D) immunoreactivity in area 17 of the human occipital cortex at low (A, B) and high (C, D) magnification. Clear astrocytic arborescence can be seen in plate D. Microphotograph E shows at high magnification a PDH immunoreactive neuron (a typical pyramidal neuron of cortical layer V, blue, ◆) of layer V and a LDH-5 immunoreactive astrocyte (protoplasmic astrocyte, brown, *) in layer IV close to the dendrite of the pyramidal neuron. Scale A, B; 500 μm; C, D: 25 μm and E: 20 μm.

**Figure 3:**
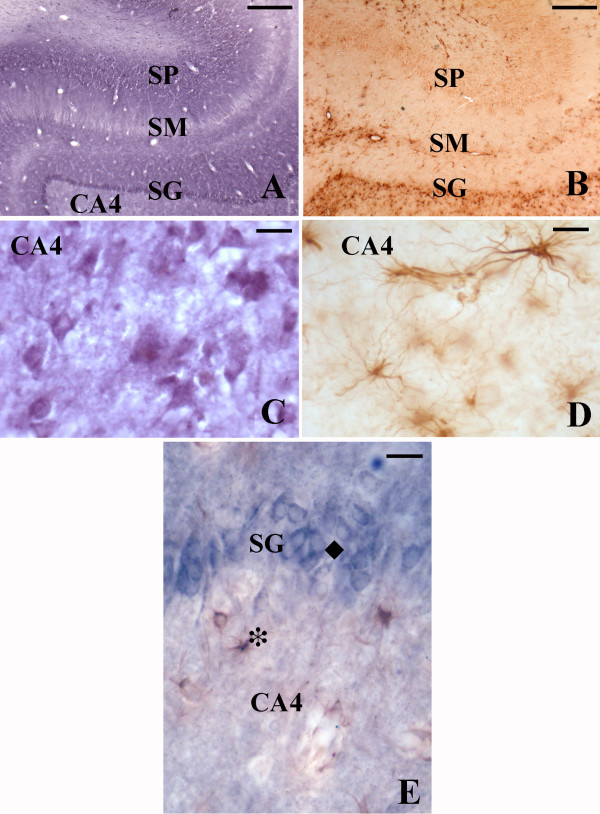
PDH like (A, C) and LDH-5 like (B, D) immunoreactivity in the human hippocampus at low (A, B) and high (C, D) magnification. Microphotograph E shows double immunolabeling (PDH, granular neuron, blue, ◆; LDH-5, protoplastmic astrocyte, brown, *). Scale A, B; 500 μm; C, D: 25 μm and E: 20 μm. Abbreviations SP: stratumpyramidale, SM: stratum moleculare and SG: stratum granulosum. Scale (A), B; 500 μm; (C), D: 25 μm and E: 20 μm.

Immunohistochemical and Western blot controls clearly showed that monoclonal antibodies (mAbs) against LDH-5 and PDH were specific for lactate dehydrogenase isoenzyme 5 and pyruvate dehydrogenase, respectively (fig 1). Figure 1A illustrates the Western Blot characterization of the anti-LDH-5 monoclonal antibody. In all cases, the antibody was specific for the monomeric form of the LDH-5 subunit whose molecular weight is 35 kDa. The antibody did not react with purified LDH-1 (fig 1A, 3), confirming its specificity for the M subunit of the enzyme. It reacted faintly with rabbit heart extracts (fig 1A, 1) that contain minute amounts of the LDH-5 subunit, and strongly with rabbit muscle extracts (fig 1A, 2), human hippocampal extracts (fig 1A, 4) and the immunogen (purified LDH-5 extracted from rabbit muscle, not shown).

Figure 1B illustrates the Western blot characterization of the mAb against PDH. On human hippocampal extracts, the antibody exclusively stained a band of approximately 43 kDa that is located in the mitochondrial fraction (fig 1B, 2) and failed to label the cytosolic fraction (fig 1B, 3). The same applied to the human occipital cortex (fig 1B, 4–6). The antibody stained a 43-kDa band from pyruvate dehydrogenase purified from porcine heart (fig 1B, 7) and did not stain purified LDH-5 extracted from rabbit muscle (fig 1B, 8, not shown as out of range of figure). Overall, the immunoreactivity at 43 kDa in human mitochondrial extracts strongly suggests that the monoclonal antibody recognizes the E1α subunit of human PDHC.

### Immunohistochemistry

The characterized monoclonal antibodies revealed patterns of immunostaining that were constant in all human brains investigated (figs. 2, 3). The two antibodies raised against two different subunits of the PDHC gave identical results.

### Human occipital cortex

In the human occipital cortex, area 17 was more specifically studied (figure 2). The PDH immunoreactivity was principally observed in layer IV, staining neuronal cell bodies (fig 2A, C). At the cellular level, virtually all neuronal bodies and dendrites were stained, whereas astrocytes were very faintly stained.

Using polyclonal antibodies (pAbs), we have previously shown that the LDH immunoreactivity of both M and H subunits is enriched in layers IV and VI of the occipital area 17 [15]. Here, as shown in figure 2B and 2D, we confirm with a monoclonal antibody our previous observation of the apparently exclusive cellular distribution of LDH-5 immunoreactivity in astrocytes throughout the white matter and in cortical layer I of the visual cortex [15]. In addition, protoplasmic astrocytes also appear in layer IV of area 17 (fig 2E) in close association with dendrites, providing a laminar staining similar to that observed by cytochrome oxidase histochemistry, immunohistochemistry and also by 2-deoxyglucose uptake studies [18,19]. In the white matter of the occipital cortex, but also in that of parietal and frontal cortices virtually all astrocytes were intensively LDH-5 immunoreactive, suggesting that white matter astrocytes in general might be principally glycolytic.

### Human hippocampus

Figure 3 shows the immunoreactivity of PDH (3A, 3C) and LDH-5 (3B, 3D) antibodies in the human hippocampus. It can clearly be observed a complementary staining of both antibodies, with intense immunostaining of LDH-5 in CA4, stratum moleculare and alveus while the stratum granulosum and the CA layers show no staining (fig 3B and 3C). PDH immunoreactivity is virtually present only in the stratum granulosum and the CA1-3 layers. Note the cellular, but also the regional metabolic compartmentalization of the two enzymes. The PDH immunolabeling was very similar to the labelling due to the pAb against LDH-1 that we have previously produced and characterized [15].

For both regions studied, a faint PDH immunoreactivity was revealed in astrocytes following longer incubation with the primary antibodies. In spite of signal amplification with TSA, no additional immunoreactivity was detected (data not shown).

## Discussion

In this article, we describe for the first time the immunohistochemical distribution of PDH and LDH-5 subunits in two regions of the human brain. Our monoclonal antibody to LDH-5 is highly specific that subunit of the enzyme and further confirms the results reported previously with the polyclonal antibody raised against rabbit muscle LDH-5 [15]. However, very recently, O'Brien *et al*. [20] report that in isolated rat brain synaptosols and cytoplasms of primary cultured neurons and astrocytes that the LDH-5 isoform (i.e. the M subunit of LDH) is present also in neurons, findings that seem to be in contradiction with the data presented here. Of course however, cell cultures do not always reflect *in vivo *nerve cell phenotypes. In the human occipital cortex and hippocampus, the 2-DG autoradiographic technique has shown that glucose is principally taken up in specific layers, i.e. the layer IV of the occipital cortex [17] and the stratum moleculare of the hippocampal formation [6]. In layer IV of the occipital cortex, a cellular compartmentalization was observed between LDH-5 and PDH immunolabeling, suggesting that astrocytes might principally produce lactate at a high rate and that the transformation of pyruvate to acetyl CoA might essentially occur in neurons. In the hippocampus, the same cellular segregation was observed, with the additional observation of a regional mismatch between both enzymes. Since 2-DG has clearly been observed to be taken up in the stratum moleculare [6], a layer enriched in LDH-5 immunoreactive astrocytes, this finding is consistent with the existence of a high glycolytic activity in astrocytes resulting in lactate production [21].

LDH-5 immunoreactivity was exclusively visualized in astrocytes in agreement with previous observation in human [15] and in astroglial cell cultures [22]. No neurons revealed any LDH-5 immunoreactivity. The LDH-5 immunoreactive astrocytes were found in regions where 2-DG uptake is known to be maximal. However, LDH-5 immunoreactive astrocytes were also present in regions where no particular 2-DG uptake has been detected, i.e. the first cortical layer of occipital area 17, the hippocampal alveus and the white matter. It is worth noting that these areas are virtually devoid of neuronal profiles. Throughout the white matter, LDH-5 labelled astrocytes were numerous, and their distribution was homogenous. In the living rat, the uptake of 2-deoxyglucose has been found to be higher in grey matter than in white matter by a factor of approximately three-fold [23], whereas cytochrome oxidase activity is about eight- to twelve-fold higher in grey matter than in white matter [24]. These observations suggest that the white matter might rely more on glycolytic metabolism than on oxidative phosphorylation and therefore produce lactate through LDH-5 subunit activity. Indeed, there is very little PDH immunoreactivity in the white matter, while the LDH-5 immunolabeling is abundant and restricted to astrocytes. Overall, the localization of the PDH immunoreactivity was similar to that previously observed [25,26] and also overlapped previous reports of cytochrome oxidase immunolabeling, although patches were not observed [18].

## Conclusion

Results reported in this article demonstrate the existence of a cellular and regional compartmentalization in two enzymes (LDH-5 and PDH) involved in glucose metabolism. At the cellular level, this compartmentalization is consistent with the notion of the existence of metabolic coupling between neurons and astrocytes, also known as the astrocyte neuron lactate shuttle hypothesis (ANLS).

Although the existence of the ANLS is still debated [27,28], evidence has come from two-photon microscopy. Kasischke *et al*. [29] measured, at a cellular resolution, the variations of fluorescence due to the variation of the free NADH/NAD^+ ^ratio. Their results shown that neuronal activity provokes oxidative metabolism in neurons closely followed by glycolytic metabolism in neighbouring astrocytes. Our present observations are fully consistent with this *ex vivo *data, whereby the intital oxidative metabolism is initiated by PDH catalysis and has a neuronal localization. The later the glycolytic activity is catalyzed by LDH-5 and is expressed in astrocytes. This late astrocytic glycolytic metabolism could be glutamate uptake stimulated [30]. As mentioned previously variation in NADH/NAD^+ ^in astrocytes may also be a key event modulating vascular tension [11]. If this is the case, one step forward has been made in understanding the cellular events underlying signals emitted in modern imaging techniques.

In conclusion, describing not only the cellular but also the regional distribution of the typically glycolytic enzyme LDH-5 in comparison to the oxidative PDH distribution provides new insight to the highly metabolic coupling of the interactions between brain cells.

## Methods

### Preparation of tissues

All experimental procedures were approved by the ethics comity of the Geneva School of Medicine (Protocol n° 04-187/Psy 04-023). The brains of 10 control patients (4 females, 6 males, mean age 72 +/- 4, post-mortem delay < 12 h) with no history of neurological, metabolic or psychiatric disorder and without histopathological lesions were obtained at autopsy from the Hospitals of the University of Geneva School of Medicine. The brains were routinely screened for histopathological lesions, using classic histological stains and immunohistochemistry procedures as described in detail elsewhere [31]. Some blocks of the hippocampal formation and occipital cortex were immediately frozen with CO_2 _for biochemical analyses, while others were fixed with 4% paraformaldehyde in 0.1 M PBS for 18 hours, then immersed in a solution containing 5% sucrose for 48 hours, and in 10% sucrose for another 48 hours before being stored at -80°C for less than a year.

### Antibodies

Wistar male rats (IFFA CREDO, Les Oncins, France) were immunised by multiple injections of LDH-5 purified from rabbit muscle (SIGMA, Buchs, Switzerland. Hybridomas were produced by fusion of splenocytes with log-phase Sp2 myeloma cells in the presence of polyethylene glycol (MW 4000; Merck, Switzerland) according to the method reported by Lövenborg [32] and described in detail elsewhere [33]. After screening by immunohistochemistry and western Blot (see below) for hybridomas producing monoclonal antibodies (mAbs) against LDH-5, positive hybridomas were propagated in a Biofarm 2000 system (Digitana, Switzerland).

The mouse mAbs respectively against the E1α and E2 subunits of human pyruvate dehydrogenase were purchased from Molecular Probes, Inc. Oregon, USA.

### Western blot analysis

The mAb against LDH-5 was characterized by immunodot, immunohistochemistry on rabbit muscle and heart sections, and by western blot analysis as described previously [15]. "Sodium-Dodecyl-Sulphate polyacrylamide gel electrophoresis" (SDS-PAGE) was performed according to the method of Laemmli [34]. Fresh tissue extracts from human hippocampus (CA) and occipital cortex (CO) were homogenized on ice with diluted sample buffer. Purified proteins were all from Sigma, Switzerland and were also diluted in sample buffer. For the mAb against PDH characterization, mitochondrial fractions and cytosolic fractions of CA and CO were extracted by successive centrifugations in 0.01 M Tris-HCl with 0.32 M sucrose according to Hamberger *et al*. [35]. Samples were heated at 100°C for 5 min, and approximately 50 μg of protein were loaded onto the gels. The extracts were then electrophoresed on polyacrylamide gels (12%) and transferred electrophoretically onto nitrocellulose membranes (Bio-Rad, Glattbrugg, Switzerland). After being blocked with non-fat dry milk and washed in phosphate buffer saline (PBS, pH 7.4), the membranes were incubated overnight with the antibodies. Detection of the bound antibodies was performed as described under "Immunohistochemistry" and/or using ECL+ with subsequent exposure to Hyperfilms (Amersham, Switzerland).

Molecular weight markers were used in all gels (Kaleidoscope and Low Range Prestained Standards from Bio-Rad).

### Immunohistochemistry

For simple immunohistochemical labelling, adjacent 20 μm cryostat (Microm HM550) cut brain sections were mounted on 0.01% poly-L-lysine (PLL, Fluka, Switzerland) coated slides and incubated overnight at 4°C in PBS containing 3% bovine serum albumin (BSA, Sigma) and 0.3% Triton X with either the anti-LDH-5 supernatant diluted 1/500 or the anti-PDHE1α diluted 1/1000 (1/10000 for the anti-PDHE2) according to the manufacturers instructions. Following incubation, sections were rinsed in PBS and incubated for one hour at room temperature with horseradish peroxidase conjugated polyclonal rabbit anti-rat IgG (for LDH-5) or anti mouse IgG (for PDHE1α and PDHE2) (DAKO, Switzerland) diluted 1/100 in PBS containing 3% BSA and 0.3% Triton X. Following PBS washes, immunoreactions were revealed for 20 min at room temperature in PBS containing 0.02% 3,3'-diaminobenzidine (DAB, Sigma) and 0.002% H_2_O_2_.

For another series of simple and double labelling, 40 μm cryostat cut free floating sections were treated as above with the difference that the PDHE1α immunolabeling was revealed in 50 mM Tris-HCl containing 0.02% DAB and 0.002% H_2_O_2 _with 0.1% nickel ammonium sulphate [36]. Double labelling was sequential, i.e. PDHE1α immunoreactivity was revealed before processing to LDH-5 immunolabelling.

An additional subset of sections were incubated for 48 hours in the two anti-PDH antibodies and processed as above or subjected to the Tyramide Signal Amplification (TSA) system (PerkinElmer life Sciences, Inc., Boston, USA) according to the manufacturer's instructions.

## Authors' contributions

JDL performed all immunohistochemical procedures and biochemical characterisation of antibodies and wrote the manuscript. YC and PhB elaborated the monoclonal antibody raised against LDH-5 and participated in the draft of the manuscript. EK and CB performed human brain autopsies and collection of specimens. LP and PJM contributed to the draft of the manuscript.
